# Supplementary material for: Neural basis for anxiety and anxiety-related physiological responses during a driving situation: an fMRI study
Source: Cereb Cortex Commun. 2022 Jun 20;3(3):tgac025. doi: 10.1093/texcom/tgac025 (PMC9279323; doi:10.1093/texcom/tgac025)
Supplement: Supplementary_Materials_R2_Final_tgac025 [file supplementary_materials_r2_final_tgac025.docx]

**Supplementary Materials**

# Supplementary Figure


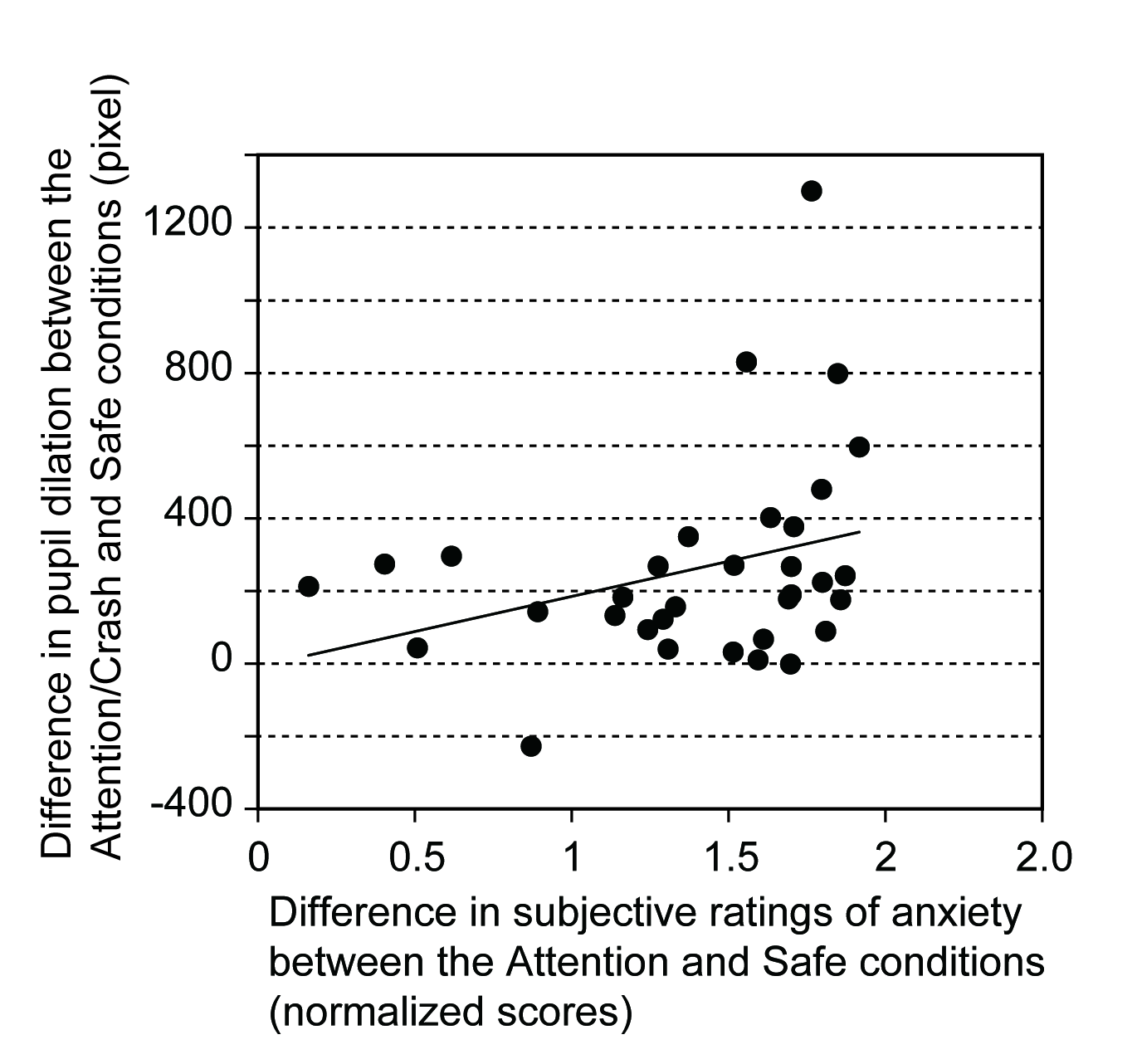


**Supplementary Fig. S1.** Pupil dilation and anxiety ratings. The correlation analysis in pupil size between the “attention/crash” and “safe” conditions, and the subjective ratings of anxiety between the “attention” and “safe” conditions. The line represents the result of a linear regression.
